# Botanical Report

**Published:** 1812-08

**Authors:** 


### BOTANICAL REPORT.

SINCE the last Botanical Report, No. 304 and 305 of the Botanical Magazine have appeared, the contents of which we shall briefly run over.

ALÖE *saponaria* (var. α) minor; the lesser or common soap-aloe: very properly a double plate, to allow of a diminished outline of the whole plant. The specific name seems to have been suggested from the spots in the leaves, which give them somewhat the resemblance of some kinds of party-colored soap.

SCILLA *non-scripta*, var. *incarnata*. A variety, according to Mr. Ker, and of Miller before him, of the common English harebells; though Linnæus had raised it into a distinct species, under the name of Hyacinthus *cernuus*, misled probably by Clusius's account of another plant, the Scilla *campanulata*, to which he had inadvertently added a figure of this plant. It happened from this mistake that Scilla *campanulata*, though well known to the Bauhins, and all the botanists of that day, was not to be found in the writings of Linnæus or his followers, till the publication of Aiton's Hortus Kewensis, when it first received the name of *campanulata*. From this we gather that Hyacinthus *cernuus*, which, with some authors, is put for S. *non-scripta*, (nutans of Dr. Smith,) with others, for S. *campanulata*, is to be expunged altogether.

Pitcairnia *integrifolia;* a new species, next to *angustifolia*, introduced from the West Indies by the late Lady Amelia Hume.

GNIDIA *imberbis;* so called by Mr. Dryander in the new edition of the Hortus Kewensis, figured in the Botanist's Repository, where it is erroneously called simplex, the name is derived from the circumstance of the eight squamulæ or nectaries being without any pubescence. Wendland mistook this species for *pinifolia*, and that again for *radiata*. The introduction of a non-descript species into a botanic garden, where those before described happen to be unknown, frequently leads to these mistakes, from botanists hastily concluding that the new plant must be one of them. We hope to see *radiata* and *pinifolia* figured in the Botanical Magazine, which will put an end to these errors.

DENTARIA *diphylla*. This species of toothwort is called pepper-root by the inhabitants of the mountains of Pennsylvania, where it is indigenous, and is used as a condiment instead of pepper or mustard. From the botanic garden in Sloane-street.

PHYTEUMA *cordata;* supposed by Dr. Sims to be Villars's plant, but totally distinct from *Nigra*, to which species *cordata* is referred by Willdenow. The plant was raised from seeds, and received from Siberia by Mr. Loddiges.

Pancratium *amœnum*. The species of Pancratium seems to be involved in considerable perplexity, which Mr. Ker has, as we think, successfully unravelled. The plant here given is supposed to be the amœnum of Salisbury, taken up by Willdenow, excluding the synonyms from Jacquin and Commelin, which belong to *caribæum*, as does also P. *fragrans*, of the new edition of Hortus Kewensis.

SCILLA *brevifolia;* Hyacinthus *brevifolius* of Thunberg and Willdenow: native of the Cape of Good Hope, whence it was introduced by Messrs. Lee and Kennedy. It is observed to come near to Hyacinthus *corymbosus* of Thunberg, given in the Magazine under the name of Massonia, but which Mr. Ker now reduces to Scilla. The author has given an amended generic character of Massonia, and we think it would not have been amiss if he had done the same for Scilla, instead of sending us to examine no less than five numbers scattered through different volumes, especially as it has generally been the custom in the Botanical Magazine, very unnecessarily indeed, to repeat the generic character under every species.

ALLIUM *cepa* (var. *β*). The tree-onion. This is commonly called in the gardens *canadense,* with which, however, it has nothing to do. Mr. Ker supposes it to be a mere bulb-bearing variety of the common onion; there are, however, two circumstances mentioned here which leave some doubt in our minds with regard to it: first, that this variety is certainly perennial, whereas the other is biennial only; secondly, it is said to be propagated not only by the bulbs produced at the top of the stalk, but likewise by offsets from the root. The first, perhaps, may be accounted for from its not producing seeds, the maturing of which destroys the life of many plants that will otherwise continue years. But it has always been remarked that the onion never produces offsets, whence it had its name of unio, and by corruption onion.

LAURUS *Diospyrus* and LAURUS *geniculata;* two nearly allied North-American deciduous Bays; the first is supposed to be the *melissæfolia* of Walter. They are both low shrubs growing in watery ground, in North Carolina; introduced by the late Mr. Fraser.

SEPTAS *globiflora;* an undescribed species of Septas, of which, however, it does not possess the character of having seven stamens, and is not distinguishable in this respect from Crassula; but, being evidently of the same genus with Septas *capensis,* Dr. Sims prefers keeping it distinct from Crassula, a genus already overgrown. From Mr. Knight's exotic nursery, Little Chelsea.

Begonia *evansiana.* This is perhaps the most beautiful species of Begonia that our collections are as yet graced with, and, being readily propagated by cuttings, will soon be very common. It was first published in the Botanist's Repository, and its specific name given it in honor of Mr. Evans, of the India-House, who spares no expence to collect rare and beautiful plants, to enrich his collection in the Commercial-road, Stepney. It appears to be cultivated in China, and is said in the Repository to have been first discovered by Mr. Evans's collector, growing in the clefts of rocks, in the island of Pulo-penang, in the East Indies; it is more probable, however, that he found it in a state of cultivation in the gardens of some of the Chinese settlers on the island. Indeed it was well known in the royal collection at Kew some years prior to this supposed discovery.

We have also another number of the BOTANIST'S REPOSITORY, containing,

CYMBIDIUM *andersonii;* supposed to be an undescribed species, a very shewy tall plant, with large yellow flowers, from the valuable collection of T. Evans, esq. of the East-India House. Mr. Andrews, in a note, appears to be very angry at a former Botanical Report, in which we were tempted to ridicule some very curious blunders in the prior number of the Repository. Had the mentioning the Cape of Good Hope instead of Cape François been the only mistake, we should have simply corrected the error, and have considered it as a mere slip of the pen, though the author seemed to have some idea that the place was at a great distance, as it is not very usual to call the going to the nearest market "travelling;" but there occurred such an assemblage of blunders in so short a space, that we could not withstand the temptation of enlivening our dry Report with a little jocularity. We must acknowledge, however, that, setting aside a little ill-humour, Mr. Andrews tells his facetious stories with a much better grace.

We can assure him, by the bye, and the whole tenor of our Reports will confirm our assertion, that we have never wished to lessen the sale of his valuable work; but, on the contrary have, on all occasions, endeavoured to promote it, as we shall continue to do; though we cannot consent to pass over his blunders, without sometimes setting him right. Could we persuade him to confine himself to his proper sphere, as a botanical draftsman, and to employ a more able pen to write his letter-press, we are convinced that the sale of his work would be increased, and his own reputation, as an author, suffer nought.

XERANTHEMUM *humile.* Introduced from the Cape of Good Hope, by Mr. Niven. The author has added a good practical direction, which we shall transcribe as applicable to the preservation of many plants. "It requires, when watered, (which should not be often,) to be thoroughly wetted, as partial watering is a general destroyer of plants, by suffering the atmosphere to exhale the humidity, before it has half penetrated to the bottom; which, by repeated wetting, rots the upper part, and leaves the roots below to starve for want of water."

CORRÆA *speciosa;* a very elegant species of a New-Holland shrub, named in honor of M. Corréa, a celebrated Portuguese botanist, now resident in Paris.

GNAPHALIUM *eximium.* A well known plant, but which does not readily flower in so great perfection as the one from which this drawing was taken.

